# Decreasing trend of blood lipid profile in type 2 diabetes: Not a promising change in HDL-C, a serial cross-sectional study

**DOI:** 10.1371/journal.pone.0293410

**Published:** 2023-10-25

**Authors:** Amirhossein Yadegar, Fatemeh Mohammadi, Soghra Rabizadeh, Alipasha Meysamie, Seyed Ali Nabipoorashrafi, Seyed Arsalan Seyedi, Alireza Esteghamati, Manouchehr Nakhjavani

**Affiliations:** 1 Endocrinology and Metabolism Research Center (EMRC), Vali-Asr Hospital, Tehran University of Medical Sciences, Tehran, Iran; 2 Department of Community and Preventive Medicine, School of Medicine, Tehran University of Medical Sciences, Tehran, Iran; 3 Cardiac Primary Prevention Research Center (CPPRC), Cardiovascular Diseases Research Institute (CVDRI), Tehran University of Medical Sciences, Tehran, Iran; Temple University School of Medicine, UNITED STATES

## Abstract

**Background:**

The prevalence of dyslipidemia in patients with type 2 diabetes (T2D) has been reported to be relatively high. The current study aimed to investigate the trend of serum lipid levels and the prevalence of dyslipidemia in patients with T2D.

**Methods:**

Data were extracted from a cohort of patients with T2D who had regular follow-ups every year for three years. TG, TC, LDL-C, HDL-C, and non-HDL-C were analyzed. The atherogenic index of plasma (AIP) was calculated using log (TG/HDL-C).

**Results:**

A total of 747 patients with T2D were included in this study, consisting of 469 (62.8%) women and 278 (37.2%) men. There was a significant downward trend in mean TG, TC, LDL-C, non-HDL-C, and AIP levels. The trend of mean HDL-C levels showed no significant change. The prevalence of high TG, high TC, high LDL-C, and high non-HDL-C significantly decreased from the first to the last visit. There was no significant change in the trend of prevalence of low HDL-C. The prevalence of high AIP significantly decreased in women and showed no significant changes in men.

**Conclusions:**

A decreasing trend was observed in the mean levels and prevalence of TG, TC, LDL-C, non-HDL-C, and AIP. HDL-C did not change significantly. The success rate in achieving a complete normal lipid profile during follow-up years was not promising and continues to be challenging.

## Introduction

The prevalence of chronic diseases such as diabetes, metabolic syndrome, hypertension, cardiovascular disease, rheumatoid arthritis, and cancer is rising due to urbanization, increasing life expectancy, sedentary lifestyle, lack of physical activity, and unhealthy eating habits [1]. Studies have shown that lifestyle modification, dietary management, physical activity, supplements, and natural components can prevent chronic diseases [2–6]. In recent years, diabetes has become one of the leading causes of morbidity and mortality, spreading worldwide at an alarming rate [7]. Diabetes increases the risk of complications such as cardiovascular disease, neuropathy, nephropathy, and retinopathy [7]. In addition to medications, lifestyle changes, and nutritional strategies can help prevent and manage diabetes [8].

Dyslipidemia is an independent risk factor in developing cardiovascular diseases (CVD), cancer, and microvascular complications of diabetes, nephropathy, and retinopathy [9–12]. The prevalence of dyslipidemia among patients with type 2 diabetes (T2D) has been reported to be more than 75% [13]. Diabetic dyslipidemia is mainly defined as high triglyceride (TG), high low-density lipoprotein cholesterol (LDL-C), or low high-density lipoprotein cholesterol (HDL-C) [14]. Statins are mainly a first-line treatment in diabetic dyslipidemia, focusing on controlling serum LDL-C levels [15]. This is due to the proven association between LDL-C and CVD [16]. Despite updated guidance on statin treatment recommendations, the success rate over time that keeps lipids in the ideal range is not reassuring. Moreover, it is essential to monitor TG or HDL-C levels as they are associated with CVD, cancer, retinopathy, and nephropathy [11, 17–20].

Timely diagnosis of adverse lipid profiles is a particular focus in patients with diabetic dyslipidemia. So, further knowledge of how lipid profile levels shift over time is required to determine the distribution of diabetic dyslipidemia and provide innovative improvement opportunities. In light of this, a few studies have been published in the past several years concerning blood lipid levels and dyslipidemia’s overall prevalence and its trend over time. In Korea, the results of a 12-year study have documented significantly increasing trends in total cholesterol (TC), LDL-C, HDL-C, and non-HDL-C among adolescents [21]. However, a downward trend in LDL-C concentration and an increasing prevalence of low HDL-C over six years have been noted among adults in China [22].

Regarding the findings of two studies during 2003–2013 and 1999–2016 in the USA, no significant changes were reported in HDL-C levels among participants. In contrast, a favorable trend was shown in non-HDL-C, TC, and median TG levels [23, 24]. Besides, research among the diabetic population taking cholesterol medication in the USA has revealed a downward linear trend for mean TC and HDL-C from 2003 through 2012 [25]. Few studies have also evaluated lipid trends in Iran. According to the results of four national health surveys during 1991–2008, trends in median cholesterol decreased in the male population. The medians of cholesterol of females had a varying trend. It decreased in ages younger than 45 but increased in ages over 45 years [26].

In addition to the inconsistencies in the current evidence, there is a lack of studies on trends in lipid profiles, primarily focusing on patients with T2D in this region. Studying the trend of serum lipid levels is worthwhile from a public health perspective to assess whether our approach to dyslipidemia had an effective role and how serum lipids change in response to management. Furthermore, previous studies mainly focused on TG, LDL-C, and HDL-C. Therefore, we conducted a study to demonstrate the trends in serum lipid levels, including TG, TC, LDL-C, HDL-C, non-HDL-C, and AIP, and the prevalence of dyslipidemia patterns in patients with T2D during follow-up years.

## Materials and methods

### Study design

This was a serial cross-sectional study using data from a cohort of patients with T2D who had regular follow-ups every year for three years in a tertiary care hospital’s diabetes clinic affiliated with the Tehran University of medical sciences between 2019 and 2021. The studied population had their routine daily activity, and we did not justify them having excess exercise. All patients received nutritional counseling at each visit by a trained dietitian. Patients who followed their regular visits every year for three years and had complete blood lipid profiles (TG, TC, LDL-C, HDL-C) were included. Therefore, 747 patients were enrolled. The population was mainly homogenous, middle class, with middle to high school education levels. They had access to healthcare facilities and insurance.

### Data collection

Demographic and socioeconomic characteristics, medications in use, and duration of diabetes variables were obtained using a structured questionnaire. Well-trained examiners conducted anthropometric measurements, including height, weight, and waist circumference. Standing height was established with a portable stadiometer (rounded to the nearest 0.1 cm). Using a calibrated balance beam scale, we measured weight in the upright position (rounded to the nearest 0.1 kg). Body mass index (BMI) is figured by dividing weight (kg) by height squared (m2) according to the Quetelet equation. We measured the waist circumference (WC) at the middle point between the lower borders of the rib cage and the iliac crest (rounded to the nearest 0.1 cm) [27]. Well-trained nurses measured blood pressure (systolic and diastolic) using a calibrated mercury sphygmomanometer. Patients who had at least 150 minutes of moderate-intensity physical activity per week were classified as physically active and others as inactive [28]. We measured fasting blood glucose (FBS), hemoglobinA1C (evaluated by high-performance liquid chromatography (HPLC)), 2-hour postprandial blood glucose (2hpp), and creatinine, as well as blood lipid profile, including TG, TC, LDL-C, and HDL-C (determined by direct enzymatic colorimetry using a Technicon RA- analyzer (Pars Azmoon, Karaj, Iran)). We calculated non-HDL-C by subtracting HDL-C from TC. The atherogenic index of plasma (AIP) was estimated according to the following equation (Log (TG/HDL-C)). The 10-year atherosclerotic cardiovascular disease (ASCVD) risk score was determined based on McClelland et al. [29] formula and categorized as low (<5%), borderline (5–7.5%), intermediate (7.5–20%), and high risk (≥20%) according to the American College of Cardiology (ACC) [30]. The estimated glomerular filtration rate (eGFR) was calculated according to the Chronic Kidney Disease Epidemiology Collaboration (CKD-EPI) equation. Under standard conditions, the patients were instructed to collect their urine in boric acid-supplied containers from 7 pm to 7 am. Urinary albumin excretion (UAE) was measured by the latex turbidimetric immunoassay method using the DAKO package (Glostrop, Denmark). This study was approved by institutional research ethics committee of Tehran University of Medical Sciences. All research was performed in accordance with the Declaration of Helsinki. Informed consent was obtained from all participants or their legal guardians.

### Treatment

Each patient has been prescribed either monotherapy or multiple-drug therapy, considering their glycemic levels. Monotherapy consisted of oral anti-diabetic drugs (OAD) or insulin, whereas treatment with two or more OAD or a combination of OAD and insulin was considered multiple-drug therapy. In this study, patients received insulin glargine or NPH insulin in combination with mealtime insulin aspart or insulin regular. Patients with hypertension were receiving relevant drugs. About 60% took angiotensin receptor blockers (ARBs) or angiotensin-converting enzyme (ACE) inhibitors. However, the entire was taking other classes of anti-hypertensive drugs containing diuretics alone (5.2%), calcium channel blockers (CCB) alone (1.6%), beta-blockers alone (1.4%), and a combination of them (nearly 30%). Because of its availability and low price, approximately 95% of all patients with dyslipidemia received atorvastatin. We did not enroll individuals receiving fibrates, ezetimibe, PCSK 9 inhibitors, or combination therapy. The medication compliances of our patients were relatively homogenous.

### Dyslipidemia definition

Diabetes was diagnosed with the American Diabetes Association (ADA) Criteria [31]. We defined dyslipidemia based on NCEP ATP III (National Cholesterol Education Program-Adult Treatment Panel III) and AHA/ACC (The American Heart Association/The American College of Cardiology) guidelines [32, 33]. The patterns were as follows: high TG (≥150 mg/dl), high total cholesterol (≥200 mg/dl), high LDL-C (≥70 mg/dl), low HDL-C (<40 mg/dl in men and <50 mg/dl in women), high non-HDL-C (≥130 mg/dl), and high AIP (>0.24) [34].

### Statistical analysis

We analyzed the data using SPSS software version 24 (SPSS, Inc.). Continuous variables were presented as means (±SD). Categorical variables were expressed as frequency and proportion. We investigated trends in serum lipid levels (TG, LDL-C, HDL-C, non-HDL-C) and dyslipidemia prevalence (high TG, high LDL-C, low HDL-C, high non-HDL-C, and high AIP) through general linear model analysis. The Kolmogorov-Smirnov test was used to analyze the normality of TG levels distribution, demonstrating a normal distribution. Results were stratified by sex. We considered a P-value lower than 0.05 statistically significant.

## Results

### Baseline characteristics

A total of 747 patients with T2D were included in this study, consisting of 469 (62.8%) women and 278 (37.2%) men. The mean age of women and men was 58.7±9.5 and 62.1±10.5 years, respectively. The mean duration of diabetes was 9.6±7.0 years in women and 9.4±7.4 years in men. Over the study period, the mean BMI increased from 29.6 to 29.9 kg/m2 in women (P-trend = 0.025) and from 27.2 to 27.9 kg/m2 in men (P-trend = 0.015). Mean WC decreased by 0.9 cm, decreasing from 96.8 to 95.9 cm (women) (P-trend = 0.106), and decreased by 0.1 cm, decreasing from 95.2 to 95.1 cm (men) (P-trend = 0.820). There was a significant downward trend in mean FBS, HbA1C, and 2hpp values in both women and men (P-value<0.001). SBP did not change significantly in both women (P-trend = 0.206) and men (P-trend = 0.691), and DBP changed significantly in women (P-trend = 0.006) and non-significantly in men (P-trend = 0.906). The 10-year ASCVD risk score showed a significant increasing trend in women (P-trend<0.001) and men (P-trend = 0.001). The prevalence of low 10-year ASCVD risk (<5%) was significantly reduced from 36.8% to 32.2% in women (P-trend = 0.002), while it was non-significantly decreased from 12.3% to 9.5% in men (P-trend = 0.090). The prevalence of borderline (5–7.5%) and intermediate (7.5–20%) 10-year ASCVD risk did not show a significant trend in women and men. The proportion of high-risk (≥20%) patients increased from 18.0% to 25.5% in women (P-trend<0.001) and from 56.8% to 60.9% in men (P-trend = 0.059) over the study period. Creatinine changed significantly in women (P-trend = 0.007) and non-significantly in men (P-trend = 0.610). The urinary albumin excretion increased from 16.5±29.6 to 17.9±60.6 mg/12h in women (P-trend = 0.737), whereas it decreased from 26.3±77.6 to 25.9±68.1 mg/12h in men (P-trend = 0.910). The baseline characteristics of the patients are shown in Table 1.

**Table 1:** The baseline characteristics of the studied patients.

		Visit 1	Visit 2	Visit 3	P-trend
**Women**					
**Age (years)**		58.7±9.5 ^a^	59.7±9.5	60.7±9.5	-
**Duration of diabetes (years)**		9.6±7.0	10.6±7.0	11.6±7.0	-
**WC (cm)**		96.3±12.0	96.9±14.9	96.2±14.4	0.923
**BMI (Kg/m2)**		29.5±4.7	30.0±6.2	29.8±4.9	0.004
**SBP (mmHg)**		132.8±20.9	132.8±20.2	134.1±17.9	0.206
**DBP (mmHg)**		79.3±12.5	77.4±10.8	77.5±10.2	0.006
**10-Year ASCVD Risk Score**		12.2±11.8	12.9±13.2	14.4±14.0	<0.001
**10-Year ASCVD**	**<%5**	36.8(32.1–41.4)	36.8(32.1–41.4)	32.2(27.7–36.7)	0.002
**Risk Score**	**%5–7.5**	10.8(7.8–13.8)	9.6(6.8–12.5)	9.1(6.4–11.9)	0.363
**categories**	**%7.5–20**	34.4(29.8–39.0)	32.0(27.5–36.5)	33.2(28.6–37.7)	0.601
**% (95% CI)**	**≥%20**	18.0(14.3–21.7)	21.6(17.7–25.6)	25.5(21.3–29.7)	<0.001
**FBS (mg/dL)**		170.1±65.9	157.3±51.8	156.1±55.6	<0.001
**2hPP (mg/dL)**		225.2±83.8	207.7±71.2	204.8±76.9	<0.001
**HbA1C (%)**		7.91±1.72	7.51±1.45	7.48±1.46	<0.001
**Creatinine (mg/dl)**		0.91±0.23	0.92±0.23	0.94±0.29	0.007
**eGFR (ml/min/1.73 m** ^**2**^ **)**		76.4±19.3	74.9±18.6	73.5±19.2	<0.001
**Medications (%)**					
**Multiple Drug Therapy**		59.1	71.2	71.6	-
**Insulin**		31.5	42.1	45.2	-
**Metformin**		86.8	97.0	88.8	-
**Any sulfonylurea**		40.3	31.8	33.5	-
**Any DPP-4 inhibitor**		1.1	1.5	5.1	-
**Atorvastatin**		83.6	95.3	96.5	-
**Rosuvastatin**		1.1	1.1	1.1	
**Antihypertensive drug**		59.7	60.5	58.8	-
**Physical activity (%)**	**Active**	38.4	39.2	39.0	0.523
**Men**					
**Age (years)**		62.1±10.5	63.1±10.5	64.1±10.5	-
**Duration of diabetes (years)**		9.4±7.4	10.4±7.4	11.4±7.4	-
**WC (cm)**		95.5±10.9	96.1±11.1	95.1±10.8	0.332
**BMI (Kg/m2)**		27.1±6.1	27.3±6.8	27.7±7.5	0.023
**SBP (mmHg)**		132.8±20.7	132.0±19.1	133.3±18.7	0.691
**DBP (mmHg)**		78.1±11.3	77.9±10.3	78.0±10.6	0.906
**10-Year ASCVD Risk Score**		25.7±18.2	25.8±18.4	27.6±19.0	0.001
**10-Year ASCVD**	**<%5**	12.3(8.2–16.5)	9.9(6.1–13.7)	9.5(5.8–13.2)	0.090
**Risk Score**	**%5–7.5**	4.9(2.2–7.7)	8.6(5.1–12.2)	6.2(3.1–9.2)	0.492
**categories**	**%7.5–20**	25.9(20.4–31.5)	22.6(17.3–27.9)	23.5(18.1–28.8)	0.356
**% (95% CI)**	**≥%20**	56.8(50.5–63.1)	58.8(52.6–65.1)	60.9(54.7–67.1)	0.059
**FBS (mg/dL)**		170.4±71.4	148.2±50.8	149.0±58.0	<0.001
**2hPP (mg/dL)**		238.5±98.1	216.7±83.2	213.5±83.2	<0.001
**HbA1C (%)**		7.86±1.92	7.34±1.53	7.39±1.55	<0.001
**Creatinine (mg/dl)**		1.12±0.29	1.12±0.31	1.11±0.30	0.610
**eGFR (ml/min/1.73 m** ^**2**^ **)**		77.8±19.3	77.7±20.5	77.4±19.9	0.704
**Medications (%)**					
**Multiple Drug Therapy**		50.5	73.0	74.2	-
**Insulin**		31.3	41.3	45.4	-
**Metformin**		78.3	94.5	87.1	-
**Any sulfonylurea**		39.3	37.9	36.7	-
**Any DPP-4 inhibitor**		1.9	2.6	9.1	-
**Atorvastatin**		82.4	96.1	96.4	-
**Rosuvastatin**		1.1	1.1	1.1	
**Antihypertensive drug**		54.3	55.8	54.0	-
**Physical activity (%)**	**Active**	46.4	47.8	47.5	0.318

^a^ Data are mean ± SD

WC:waist circumference; BMI:body mass index; SBP:systolic blood pressure; DBP:diastolic blood pressure; FBS:fast blood sugar; 2hPP:2-hour postprandial

### Trends in means of serum lipid profile levels

**Fig 1:**
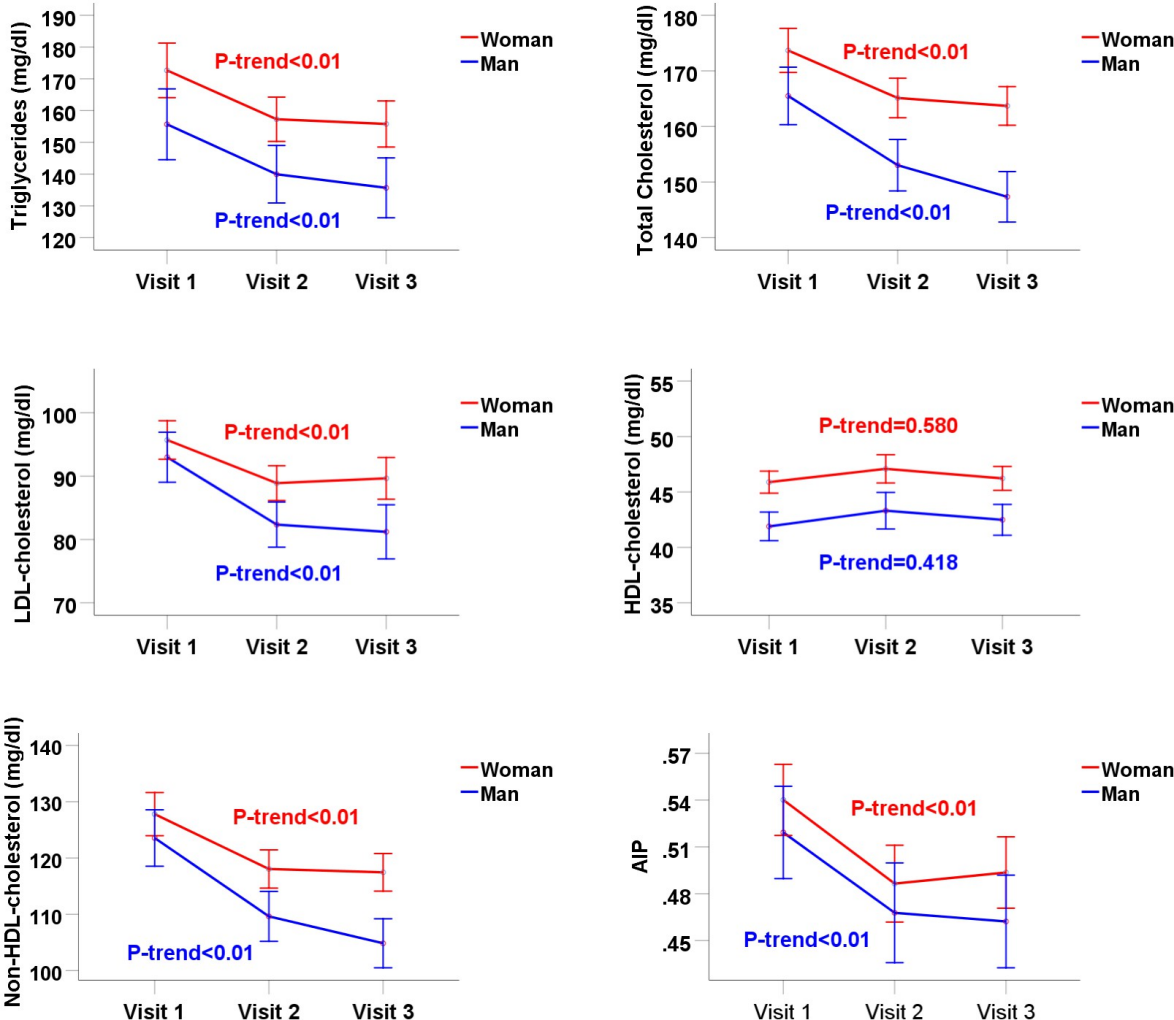
Trends in mean serum lipid profiles in patients with type 2 diabetes. TG: triglyceride; LDL: low-density lipoprotein; HDL: high-density lipoprotein; non-HDL: non-high-density lipoprotein (Total cholesterol minus HDL-C); AIP: atherogenic index of plasma (log (TG/HDL-C)).

Mean TG levels were significantly decreased by 16.9 mg/dl, decreasing from 172.6 to 155.7 mg/dl (women) (P-value<0.001), and by 20 mg/dl, decreasing from 155.6 to 135.6 mg/dl (men) (P-trend = 0.001) (Table 2). There was also a significant downward trend in mean levels of TC, LDL-C, non-HDL-C, and AIP, whereas mean HDL-C levels showed no significant trend in both women and men (Fig 1). Mean TC levels decreased by 9.9 mg/dl, decreasing from 173.6 to 163.7 mg/dl (women) (P-value<0.001), and by 18.1 mg/dl, decreasing from 165.4 to 147.3 mg/dl (men) (P-value<0.001). Mean LDL-C levels decreased from 95.7 to 89.6 mg/dl in women (P-trend = 0.005) and 92.9 to 81.2 mg/dl in men (P-value<0.001). Mean non-HDL-C levels decreased from the first to the last visit, from 127.7 to 117.4 mg/dl in women (P-value<0.001) and from 123.5 to 104.8 mg/dl in men (P-value<0.001). Mean AIP levels decreased from 0.54 to 0.49 in women (P-value<0.001) and from 0.51 to 0.46 in men (P-value<0.001). Mean HDL-C levels increased by 0.4 mg/dl, rising from 45.8 to 46.2 mg/dl (women) (P-trend = 0.580), and by 0.6 mg/dl, rising from 41.8 to 42.4 mg/dl (men) (P-trend = 0.418) (Table 2).

**Table 2:** Trends in mean serum lipid profiles in patients with type 2 diabetes.

	Visit 1	95% CI	Visit 2	95% CI	Visit 3	95% CI	P-trend
**Women**							
**TG (mg/dl)**	172.6±4.4 ^a^	164.1–181.2	157.2±3.6	150.3–164.2	155.7±3.7	148.5–163.0	<0.001
**TC (mg/dl)**	173.6±2.0	169.7–177.6	165.1±1.8	161.6–168.7	163.7±1.8	160.2–167.2	<0.001
**LDL-C (mg/dl)**	95.7±1.5	92.7–98.7	88.8±1.4	86.2–91.6	89.6±1.7	86.4–93.0	0.005
**HDL-C (mg/dl)**	45.8±0.5	44.9–46.9	47.0±0.6	45.8–48.4	46.2±0.5	45.2–47.3	0.580
**Non-HDL-C (mg/dl)**	127.7±2.0	124.0–131.6	118.0±1.7	114.6–121.4	117.4±1.7	114.1–120.8	<0.001
**AIP**	0.54±0.01	0.52–0.56	0.48±0.01	0.46–0.51	0.49±0.01	0.47–0.52	<0.001
**Men**							
**TG (mg/dl)**	155.6±5.7	144.5–166.8	139.9±4.6	130.9–149.0	135.6±4.8	126.2–145.1	0.001
**TC (mg/dl)**	165.4±2.6	160.3–170.7	153.0±2.4	148.4–157.7	147.3±2.3	142.8–151.9	<0.001
**LDL-C (mg/dl)**	92.9±2.0	89.0–96.9	82.3±1.8	78.8–85.9	81.2±2.2	76.9–85.5	<0.001
**HDL-C (mg/dl)**	41.8±0.7	40.6–43.2	43.3±0.8	41.7–45.0	42.4±0.7	41.1–43.9	0.418
**Non-HDL-C (mg/dl)**	123.5±2.6	118.5–128.6	109.6±2.3	105.2	104.8±2.2	100.5–109.2	<0.001
**AIP**	0.51±0.01	0.49–0.55	0.46±0.01	0.44–0.50	0.46±0.01	0.43–0.49	<0.001

^a.^ Data are mean ± SE

TG: triglyceride; TC: total cholesterol; LDL: low-density lipoprotein; HDL: high-density lipoprotein non-HDL: non-high-density lipoprotein (TC minus HDL-C); AIP: atherogenic index of plasma (log (TG/HDL-C))

### Trends in the prevalence of dyslipidemia

**Fig 2:**
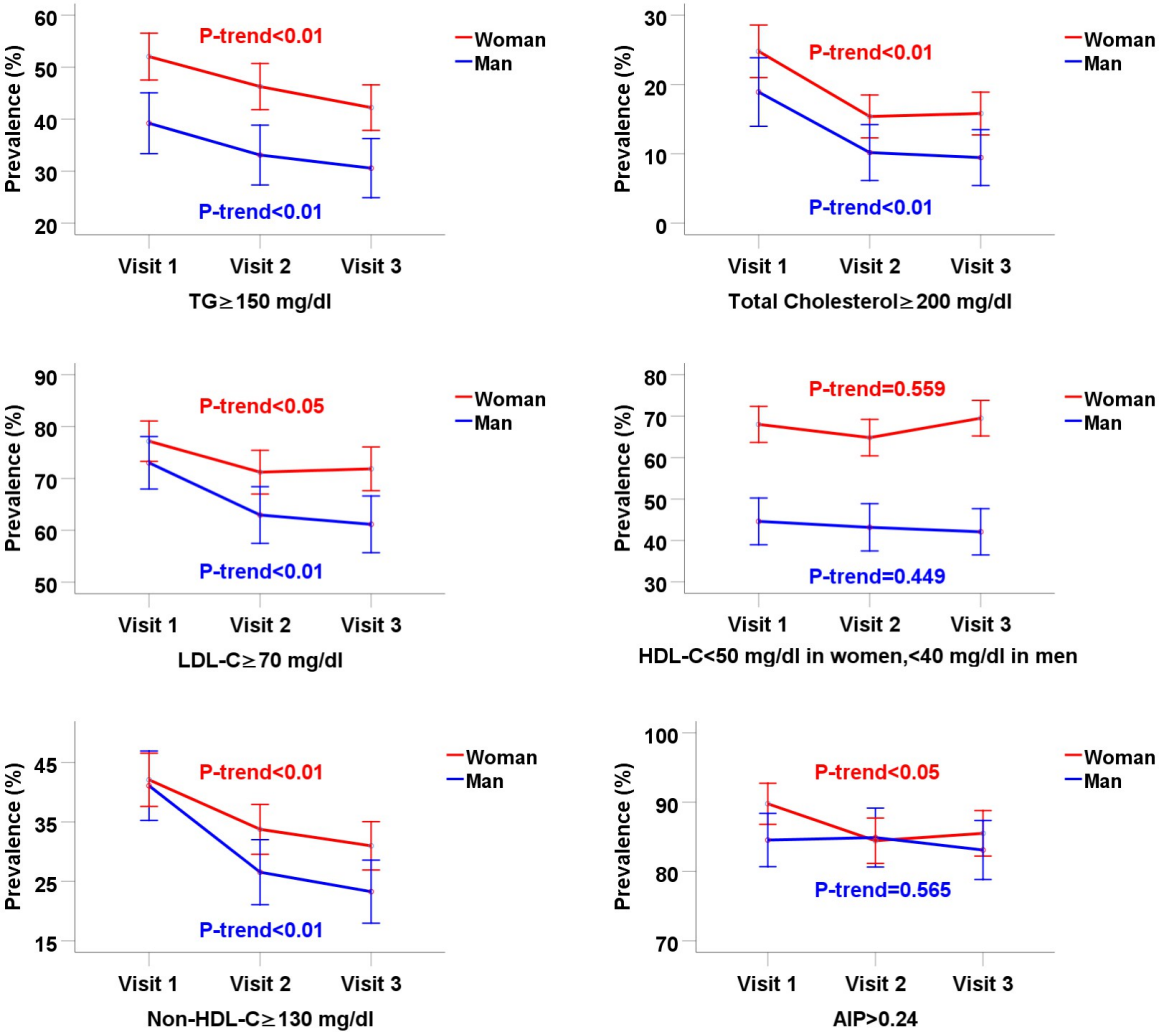
Trends in the prevalence of dyslipidemia in patients with type 2 diabetes. TG: triglyceride; LDL: low-density lipoprotein; HDL: high-density lipoprotein; non-HDL: non-high-density lipoprotein (Total cholesterol minus HDL-C); AIP: atherogenic index of plasma (log (TG/HDL-C)).

The prevalence of high TG (≥150 mg/dl) decreased over the study period, decreasing from 52.0% to 42.2% in women (P-value<0.001) and from 39.2% to 30.6% in men (P-trend = 0.008) (Table 3). The prevalence of high TC (≥200 mg/dl), high LDL-C (≥70 mg/dl), and high non-HDL-C (≥130 mg/dl) significantly decreased from the first to the last visit, whereas there was no significant trend in the prevalence of low HDL-C (<50 mg/dl in women, <40 mg/dl in men) in both women and men (Fig 2). The prevalence of high TC decreased from 24.8% to 15.8% in women (P-value<0.001) and 18.9% to 9.4% in men (P-value<0.001). The prevalence of high LDL-C decreased from 77.2% to 71.9% in women (P-trend = 0.028) and from 73.0% to 61.2% in men (P-trend = 0.001). The prevalence of high non-HDL-C decreased by 11.2%, decreasing from 42.1% to 30.9% (women) (P-value<0.001) and by 17.8%, decreasing from 41.1% to 23.3% (men) (P-value<0.001). The prevalence of low HDL-C increased from 68.0% to 69.5% in women (P-trend = 0.559) and decreased from 44.6% to 42.1% in men (P-trend = 0.449). The prevalence of high AIP (>0.24) significantly decreased from 89.8% to 85.5% in women (P-trend = 0.023) and non-significantly reduced from 84.5% to 83.1% in men (P-trend = 0.565) (Table 3).

**Table 3:** Trends in the prevalence of dyslipidemia in patients with type 2 diabetes.

	Visit 1	95% CI	Visit 2	95% CI	Visit 3	95% CI	P-trend
**Women**							
**TG ≥150 mg/dl**	%52.0 ^a^	%47.5–56.5	%46.3	%41.9–50.7	%42.2	%37.8–46.6	<0.001
**Total Cholesterol ≥200 mg/dl**	%24.8	%21.0–28.6	%15.4	%12.3–18.5	%15.8	%12.7–18.9	<0.001
**LDL-C ≥70mg/dl**	%77.2	%73.3–81.1	%71.2	%67.0–75.4	%71.9	%67.6–76.1	0.028
**HDL-C (<40 in men, <50 in women)**	%68.0	%63.7–72.4	%64.8	%60.4–69.2	%69.5	%65.2–73.8	0.559
**Non-HDL-C ≥130 mg/dl**	%42.1	%37.6–46.6	%33.8	%29.6–38.0	%30.9	%26.9–35.1	<0.001
**AIP >0.24**	%89.8	%86.8–92.7	%84.4	%81.2–87.7	%85.5	%82.2–88.8	0.023
**Men**							
**TG ≥150 mg/dl**	%39.2	%33.4–45.0	%33.1	%27.3–38.9	%30.6	%24.9–36.3	0.008
**Total Cholesterol ≥200 mg/dl**	%18.9	%14.0–23.9	%10.2	%6.1–14.2	%9.4	%5.4–13.5	<0.001
**LDL-C ≥70mg/dl**	%73.0	%68.0–78.1	%62.9	%57.5–68.4	%61.2	%55.7–66.6	0.001
**HDL-C (<40 in men, <50 in women)**	%44.6	%39.0–50.2	%43.2	%37.5–48.9	%42.1	%36.5–47.7	0.449
**Non-HDL-C ≥130 mg/dl**	%41.1	%35.2–46.9	%26.5	%21.1–32.0	%23.3	%18.0–28.6	<0.001
**AIP >0.24**	%84.5	%80.7–88.4	%84.9	%80.6–89.1	%83.1	%78.8–87.3	0.565

^a.^ Data are prevalence (%)

TG: triglyceride; LDL: low-density lipoprotein; HDL: high-density lipoprotein non-HDL: non-high-density lipoprotein (Total cholesterol minus HDL-C); AIP: atherogenic index of plasma (log (TG/HDL-C))

### The success rate for blood lipid goals achievement

**Fig 3:**
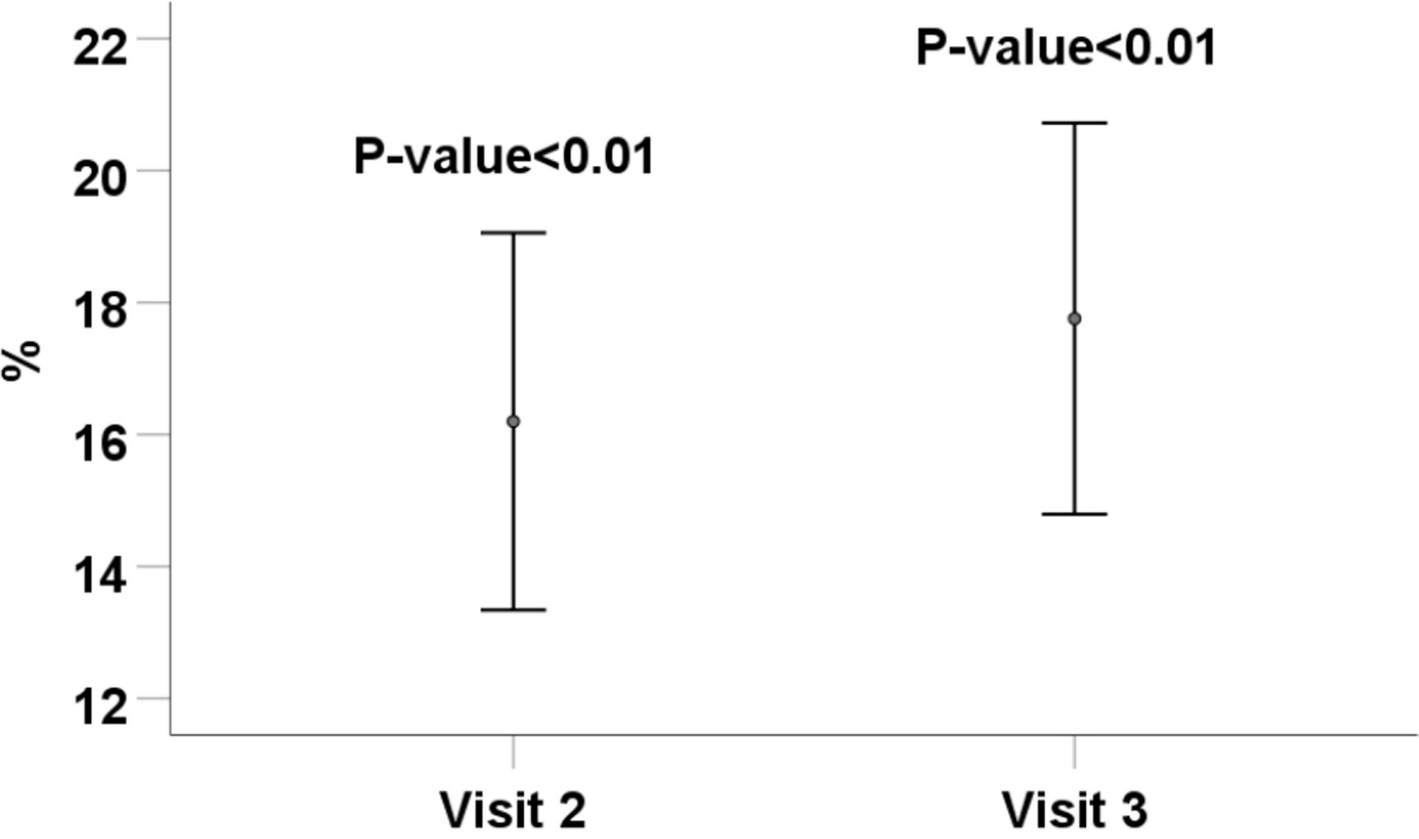
The success rate for blood lipid goals achievement, including TG<150 mg/dl, TC<200 mg/dl, LDL-C<70 mg/dl, and non-HDL-C<130 mg/dl. TG: triglyceride; TC: total cholesterol; LDL: low-density lipoprotein; non-HDL: non-high-density lipoprotein (TC minus HDL-C).

Seven hundred and twenty-four patients (96.9%) out of 747 had at least one lipid abnormality in visit 1. The success rate for blood lipid goals achievement, including TG<150 mg/dl, TC<200 mg/dl, LDL-C<70 mg/dl, and non-HDL-C<130 mg/dl was 16.2% (95% CI: 13.3–19.1%) in visit 2 (P-value<0.001) and 17.8% (95% CI: 14.8–20.7%) in visit 3 (P-value<0.001) (Fig 3).

## Discussion

According to a study published in 2011, 11.9% of individuals aged 25–70 years had diabetes in Iran [35]. The prevalence of dyslipidemia among patients with T2D was reported to be near 75% [13]. The abnormal levels of serum lipids can lead to cardiovascular disease, cancer, and microvascular complications of diabetes [9–12]. Hence, we investigated trends in means of serum lipid profile levels and prevalence of dyslipidemia in patients with T2D over three years. This study shows changes in serum lipid levels and the prevalence of dyslipidemia during follow-up visits and can elucidate whether the management was adequate for the patients.

In this study, mean serum TG, TC, LDL-C, and non-HDL-C levels had a significant downward trend among patients with T2D (p-trend<0.01). Our findings are consistent with studies conducted in this region. Among Iranian patients with diabetes, mean serum TC levels significantly decreased from 1999–2018 [36]. Also, mean serum TG, LDL-C, and non-HDL-C levels significantly reduced from 2007–2016 among patients with diabetes in Iran [37]. Among diabetic adults in the USA, mean TC significantly decreased from 2003–2012 [25]. Significant decreases were reported in non-HDL-C and TC levels in patients with diabetes mellitus without atherosclerotic cardiovascular disease in the USA from 1999–2016 [23]. Additionally, an analysis of patients with diabetes from 2007 to 2018 in the USA showed that TG, TC, and LDL-C decreased significantly [38].

We also found favorable trends in the prevalence of high TG, high TC, high LDL-C, and high non-HDL-C (P-trend<0.01). Among adults in China, the prevalence of high LDL-C significantly decreased from 2009–2015; high TC and high TG showed no significant changes in this study. They suggest several reasons for these changes, including improvement of economic and health awareness and more investment in medical and health services [22]. The prevalence of high TG declined in the adult population in the USA from 1999–2010 [39]. Increasing trends in the prevalence of high TC and high LDL-C and no significant changes in the prevalence of high TG and high non-HDL-C were reported in Korean adolescents. Dietary fat or dairy consumption changes have been reported as possible reasons for this trend [21]. Among adults with newly diagnosed T2D in China from 2003–2012, the prevalence of TC had significantly increased. However, no significant changes were observed in the trend of high TG and high LDL-C prevalence [40].

The significant downward trends in the prevalence of dyslipidemia patterns and the mean values of TG, TC, LDL-C, and non-HDL-C in this study may be caused by lifestyle modification or lipid-lowering drugs. Also, diabetes management and improved blood glucose control may have facilitated this process [41]. Due to the availability and low price of lipid-lowering medications, adults with T2D had a high rate of adherence to statins in the current study. Regarding enhanced access to social media, socioeconomic groups’ awareness of medical issues has increased in past decades worldwide [42]. As a result, dyslipidemia has received more attention, and patients are more interested in their lipid profile evaluation during follow-up visits.

Earlier studies have shown that low HDL-C is common among the Iranian population, both with and without diabetes [43]. On the other hand, impaired HDL-C serum levels have been shown to elevate the risk of cardiovascular disease and other conditions, including cancer [44, 45]. In this study, mean serum HDL-C levels did not significantly change in women and men (The p-trend in men and women was 0.418 and 0.580, respectively). Studies on trends in HDL-C levels are controversial. In US adults, mean HDL-C remained constant over the ten years [24]. Also, no significant changes were noted in HDL-C levels in adults with diabetes in the USA from 1999–2016 [23]. In addition, Chen et al. showed no significant changes in HDL-C levels in patients with diabetes in the USA from 2007–2018 [38]. However, Mercado et al. reported that mean HDL-C significantly decreased among diabetic adults taking cholesterol-lowering medication in the USA [25]. Malekzadeh et al. reported an adverse trend in HDL-C levels among Iranian patients with diabetes [37].

In this study, no significant changes were observed in the prevalence of low HDL-C over the study period. As with trends in HDL-C levels, reports on trends in the prevalence of low HDL-C are arguable. An overall decline in the prevalence of low HDL-C among adults in the general population in the USA has been observed [39]. Among newly diagnosed T2D patients in China, the prevalence of low HDL-C increased significantly [40]. Moreover, an increasing prevalence of low HDL-C among adults in China has been reported [22]. In contrast, the prevalence of low HDL-C decreased significantly among adolescents in Korea from 2007–2018 [21].

McTaggart et al. reported increased HDL-C levels by 4–10% after statin treatment in a review article published in 2008 [46]. Even a 2.8% decrease in HDL-C after statin treatment has been reported [47]. A study among European patients treated for dyslipidemia showed that despite lipid-modifying treatment, the prevalence of low HDL-C was more than 30% [48]. Vaisar et al. showed that HDL-C is a complex mixture of proteins that function in lipid metabolism (22 proteins), proteinase inhibition (8 proteins), complement activation (6 proteins), and the acute-phase response (23 proteins) [49]. HDL-C is a relatively stable molecule and has fewer fluctuations. Impaired HDL-C function has been reported in T2D [50]. Unlike other serum lipid profile components, therapeutic efforts aim to increase HDL-C, although the published literature does not show promising success in this case [23–25, 37].

Also, we investigated the trend of AIP, which, despite its high prevalence in patients with T2D [51], has not been studied yet. AIP has been shown to predict cardiovascular risk better than its components alone (TG, HDL-C) [34]. There was a significant downward trend in the mean values of AIP in women and men. The prevalence of high AIP in women decreased significantly. No significant change was observed in the prevalence of high AIP in men. Although mean levels and prevalence have decreased, values remained high, and AIP should therefore be considered a critical lipid disorder.

In the current study, the 10-year ASCVD risk score showed a significantly increasing trend in women and men. The prevalence of low 10-year ASCVD risk (<5%) was significantly reduced in women, while it was non-significantly decreased in men. However, the proportion of high-risk (≥20%) patients increased significantly in women and non-significantly in men over the study period. Our findings are consistent with previous studies [52, 53]. Ling et al. showed that the prevalence of high ASCVD risk had an increasing trend in patients with T2D [52]. Another study found that the ASCVD risk score and the proportion of high-risk participants increased from 1999 to 2018 in the United States [53].

The success rate of dyslipidemia treatment was also assessed in the current study. A total of 17.8% of patients achieved blood lipid goals, including TG<150 mg/dl, TC<200 mg/dl, LDL-C<70 mg/dl, and non-HDL-C<130 mg/dl over the study period. Studies on achieving ABC goals in diabetes have focused primarily on LDL-C control [54]. The overall control rate of LDL-C and non-HDL-C among patients with T2D in China has been reported at 43.1% and 19.8%, respectively [55]. Among patients with T2D in Iran, the achievement of LDL-C ≤100 mg/dl was 48.1% [56]. In the current study, patients were from a tertiary care hospital. This may be a factor in the low success rate in the present study. Also, achieving a normal lipid profile, including TG<150, TC<200 mg/dl, LDL-C<70 mg/dl, and non-HDL-C<130 mg/dl, may be difficult.

A key strength of the current study was that in addition to TG, LDL-C, and HDL-C, which are usually emphasized as diabetic dyslipidemia, we also studied the trends of non-HDL-C and AIP.

There are several limitations to this study:

1. The study period was only three years.
2. Patients who were followed up on their regular visits for three years may have been more compliant with treatment and lifestyle modification than patients who did not.
3. The number of patients was relatively small.

## Conclusions

A decreasing trend was observed in the mean level and prevalence of TG, TC, LDL-C, non-HDL-C, and AIP. HDL-C did not change significantly. The success rate in achieving a complete normal lipid profile during follow-up years was not promising and continues to be challenging.

## Supporting information

S1 Dataset (XLSX)

## References

[1] Paganini-Hill A, Ross RK, Henderson BE. Prevalence of chronic disease and health practices in a retirement community. Journal of Chronic Diseases. 1986;39(9):699–707. doi: 10.1016/0021-9681(86)90153-0 3734024

[2] Mansouri M, Pahlavani N, Sharifi F, Varmaghani M, Shokri A, Yaghubi H, et al. Dairy Consumption in Relation to Hypertension Among a Large Population of University Students: The MEPHASOUS Study. Diabetes Metab Syndr Obes. 2020;13:1633–42. doi: 10.2147/DMSO.S248592 32523363 PMC7234968

[3] Nattagh-Eshtivani E, Pahlavani N, Ranjbar G, Gholizadeh Navashenaq J, Salehi-Sahlabadi A, Mahmudiono T, et al. Does propolis have any effect on rheumatoid arthritis? A review study. Food Science & Nutrition. 2022;10(4):1003–20. doi: 10.1002/fsn3.2684 35432965 PMC9007309

[4] Nattagh-Eshtivani E, Gheflati A, Barghchi H, Rahbarinejad P, Hachem K, Shalaby MN, et al. The role of Pycnogenol in the control of inflammation and oxidative stress in chronic diseases: Molecular aspects. Phytotherapy Research. 2022;36(6):2352–74. doi: 10.1002/ptr.7454 35583807

[5] Pahlavani N, Khayyatzadeh SS, Banazadeh V, Bagherniya M, Tayefi M, Eslami S, et al. Adherence to a Dietary Approach to Stop Hypertension (DASH)-Style in Relation to Daytime Sleepiness. Nat Sci Sleep. 2020;12:325–32. doi: 10.2147/NSS.S246991 32607032 PMC7292369

[6] Rabizadeh S, Heidari F, Karimi R, Rajab A, Rahimi-Dehgolan S, Yadegar A, et al. Vitamin C supplementation lowers advanced glycation end products (AGEs) and malondialdehyde (MDA) in patients with type 2 diabetes: A randomized, double-blind, placebo-controlled clinical trial. Food Science & Nutrition.n/a(n/a). doi: 10.1002/fsn3.3530 37823170 PMC10563761

[7] Aynalem SB, Zeleke AJ. Prevalence of Diabetes Mellitus and Its Risk Factors among Individuals Aged 15 Years and Above in Mizan-Aman Town, Southwest Ethiopia, 2016: A Cross Sectional Study. Int J Endocrinol. 2018;2018:9317987. doi: 10.1155/2018/9317987 29853887 PMC5944196

[8] Afable A, Karingula NS. Evidence based review of type 2 diabetes prevention and management in low and middle income countries. World J Diabetes. 2016;7(10):209–29. doi: 10.4239/wjd.v7.i10.209 27226816 PMC4873312

[9] Miller M. Dyslipidemia and cardiovascular risk: the importance of early prevention. QJM: An International Journal of Medicine. 2009;102(9):657–67. doi: 10.1093/qjmed/hcp065 19498039 PMC2729130

[10] Mayengbam SS, Singh A, Pillai AD, Bhat MK. Influence of cholesterol on cancer progression and therapy. Translational Oncology. 2021;14(6):101043. doi: 10.1016/j.tranon.2021.101043 33751965 PMC8010885

[11] Kawanami D, Matoba K, Utsunomiya K. Dyslipidemia in diabetic nephropathy. Renal Replacement Therapy. 2016;2(1):1–9.

[12] Jeng C-J, Hsieh Y-T, Yang C-M, Yang C-H, Lin C-L, Wang I-J. Diabetic retinopathy in patients with dyslipidemia: development and progression. Ophthalmology Retina. 2018;2(1):38–45. doi: 10.1016/j.oret.2017.05.010 31047300

[13] Athyros VG, Doumas M, Imprialos KP, Stavropoulos K, Georgianou E, Katsimardou A, et al. Diabetes and lipid metabolism. Hormones. 2018;17(1):61–7. doi: 10.1007/s42000-018-0014-8 29858856

[14] Chehade JM, Gladysz M, Mooradian AD. Dyslipidemia in type 2 diabetes: prevalence, pathophysiology, and management. Drugs. 2013;73(4):327–39. doi: 10.1007/s40265-013-0023-5 23479408

[15] Maki KC, Dicklin MR, Baum SJ. Statins and diabetes. Cardiology Clinics. 2015;33(2):233–43. doi: 10.1016/j.ccl.2015.02.004 25939296

[16] Silverman MG, Ference BA, Im K, Wiviott SD, Giugliano RP, Grundy SM, et al. Association between lowering LDL-C and cardiovascular risk reduction among different therapeutic interventions: a systematic review and meta-analysis. Jama. 2016;316(12):1289–97. doi: 10.1001/jama.2016.13985 27673306

[17] Harchaoui K, Visser M, Kastelein J, Stroes E, Dallinga-Thie G. Triglycerides and cardiovascular risk. Current cardiology reviews. 2009;5(3):216–22. doi: 10.2174/157340309788970315 20676280 PMC2822144

[18] Chang Y-C, Wu W-C. Dyslipidemia and diabetic retinopathy. The review of diabetic studies: RDS. 2013;10(2–3):121. doi: 10.1900/RDS.2013.10.121 24380088 PMC4063092

[19] Notarnicola M, Altomare DF, Correale M, Ruggieri E, D’Attoma B, Mastrosimini A, et al. Serum lipid profile in colorectal cancer patients with and without synchronous distant metastases. Oncology. 2005;68(4–6):371–4. doi: 10.1159/000086977 16020965

[20] Siemianowicz K, Gminski J, Stajszczyk M, Wojakowski W, Goss M, Machalski M, et al. Serum total cholesterol and triglycerides levels in patients with lung cancer. International journal of molecular medicine. 2000;5(2):201–6. doi: 10.3892/ijmm.5.2.201 10639602

[21] Jeong D-Y, Kim S-H, Seo MY, Kang SY, Park MJ. Trends in Serum Lipid Profiles Among Korean Adolescents, 2007–2018. Diabetes, Metabolic Syndrome and Obesity: Targets and Therapy. 2021;14:4189. doi: 10.2147/DMSO.S326070 34675571 PMC8504865

[22] Yuan X, Ni W, Wang R, Chi H, Sun Y, Lv D, et al. 6-Year trends in lipids among adults in Shenzhen, China. Journal of Public Health. 2020;42(4):e468–e76. doi: 10.1093/pubmed/fdz113 31728508

[23] Vega GL, Wang J, Grundy SM. Temporal decline in non–high-density lipoprotein cholesterol in subjects with diabetes mellitus without atherosclerotic cardiovascular disease. Journal of Clinical Lipidology. 2020;14(4):425–30. doi: 10.1016/j.jacl.2020.04.007 32467016

[24] Palmer MK, Toth PP. Trends in lipids, obesity, metabolic syndrome, and diabetes mellitus in the United States: An NHANES analysis (2003‐2004 to 2013‐2014). Obesity. 2019;27(2):309–14. doi: 10.1002/oby.22370 30677260

[25] Mercado CI, Gregg E, Gillespie C, Loustalot F. Trends in lipid profiles and descriptive characteristics of US adults with and without diabetes and cholesterol-lowering medication use—National Health and Nutrition Examination Survey, 2003–2012, United States. PLoS One. 2018;13(3):e0193756. 29509776 10.1371/journal.pone.0193756 PMC5839584

[26] Hosseini M, Yousefifard M, Taslimi S, Sohanaki H, Nourijelyani K, Asgari F, et al. Trend of blood cholesterol level in iran: results of four national surveys during 1991–2008. Acta Medica Iranica. 2013:642–51. 24338197

[27] Shi W, Neubeck L, Gallagher R. Measurement matters: A systematic review of waist measurement sites for determining central adiposity. Collegian. 2017;24(5):513–23.

[28] Colberg SR, Sigal RJ, Yardley JE, Riddell MC, Dunstan DW, Dempsey PC, et al. Physical activity/exercise and diabetes: a position statement of the American Diabetes Association. Diabetes care. 2016;39(11):2065. doi: 10.2337/dc16-1728 27926890 PMC6908414

[29] McClelland RL, Jorgensen NW, Budoff M, Blaha MJ, Post WS, Kronmal RA, et al. 10-Year Coronary Heart Disease Risk Prediction Using Coronary Artery Calcium and Traditional Risk Factors: Derivation in the MESA (Multi-Ethnic Study of Atherosclerosis) With Validation in the HNR (Heinz Nixdorf Recall) Study and the DHS (Dallas Heart Study). Journal of the American College of Cardiology. 2015;66(15):1643–53. doi: 10.1016/j.jacc.2015.08.035 26449133 PMC4603537

[30] Arps K, Blumenthal R, Martin S. New aspects of the risk assessment guidelines: practical highlights, scientific evidence and future goals. American College of Cardiology. 2018.

[31] Committee ADAPP. 2. Classification and Diagnosis of Diabetes: Standards of Medical Care in Diabetes—2022. Diabetes Care. 2021;45(Supplement_1):S17-S38. 10.2337/dc22-S002 34964875

[32] Detection NCEPEPo, Adults ToHBCi. Third report of the National Cholesterol Education Program (NCEP) Expert Panel on detection, evaluation, and treatment of high blood cholesterol in adults (Adult Treatment Panel III): The Program; 2002. 12485966

[33] Grundy SM, Stone NJ, Bailey AL, Beam C, Birtcher KK, Blumenthal RS, et al. 2018 AHA/ACC/AACVPR/AAPA/ABC/ACPM/ADA/AGS/APhA/ASPC/NLA/PCNA guideline on the management of blood cholesterol: a report of the American College of Cardiology/American Heart Association Task Force on Clinical Practice Guidelines. Journal of the American College of Cardiology. 2019;73(24):e285–e350. 30423393 10.1016/j.jacc.2018.11.003

[34] Dobiasova M. AIP—atherogenic index of plasma as a significant predictor of cardiovascular risk: from research to practice. Vnitrni lekarstvi. 2006;52(1):64–71. 16526201

[35] Mirzaei M, Rahmaninan M, Mirzaei M, Nadjarzadeh A. Epidemiology of diabetes mellitus, pre-diabetes, undiagnosed and uncontrolled diabetes in Central Iran: results from Yazd health study. BMC public health. 2020;20(1):1–9. 32013917 10.1186/s12889-020-8267-y PMC6998152

[36] Koohi F, Kohansal K, Naz MSG, Derakhshan S, Azizi F, Khalili D. The trend of 10-year cardiovascular risk among diabetic and non-diabetic participants in Tehran Lipid and glucose study: 1999–2018. BMC public health. 2022;22(1):1–9. 35346132 10.1186/s12889-022-12981-9 PMC8961927

[37] Malekzadeh H, Lotfaliany M, Ostovar A, Hadaegh F, Azizi F, Yoosefi M, et al. Trends in cardiovascular risk factors in diabetic patients in comparison to general population in Iran: findings from National Surveys 2007–2016. Scientific reports. 2020;10(1):1–10. 32678170 10.1038/s41598-020-68640-9 PMC7366682

[38] Chen T, Wang Z, Xie J, Xiao S, Liu N. Trends in lipid profiles and control of LDL-C among adults with diabetes in the United States: An analysis of NHANES 2007–2018. Nutrition, Metabolism and Cardiovascular Diseases. 2023;33(7):1367–76. doi: 10.1016/j.numecd.2023.04.012 37156669

[39] Beltrán-Sánchez H, Harhay MO, Harhay MM, McElligott S. Prevalence and trends of metabolic syndrome in the adult US population, 1999–2010. Journal of the American College of Cardiology. 2013;62(8):697–703. 23810877 10.1016/j.jacc.2013.05.064 PMC3756561

[40] Tian J, Chen H, Jia F, Yang G, Li S, Li K, et al. Trends in the levels of serum lipids and lipoproteins and the prevalence of dyslipidemia in adults with newly diagnosed type 2 diabetes in the Southwest Chinese Han Population during 2003–2012. International Journal of Endocrinology. 2015;2015. doi: 10.1155/2015/818075 26089896 PMC4451154

[41] Schofield JD, Liu Y, Rao-Balakrishna P, Malik RA, Soran H. Diabetes dyslipidemia. Diabetes therapy. 2016;7(2):203–19. doi: 10.1007/s13300-016-0167-x 27056202 PMC4900977

[42] Kass-Hout TA, Alhinnawi H. Social media in public health. British medical bulletin. 2013;108(1). 10.1093/bmb/ldt028 24103335

[43] Aryan Z, Mahmoudi N, Sheidaei A, Rezaei S, Mahmoudi Z, Gohari K, et al. The prevalence, awareness, and treatment of lipid abnormalities in Iranian adults: Surveillance of risk factors of noncommunicable diseases in Iran 2016. Journal of clinical lipidology. 2018;12(6):1471–81. e4. doi: 10.1016/j.jacl.2018.08.001 30195823

[44] Bruckert E, Hansel B. HDL‐c is a powerful lipid predictor of cardiovascular diseases. International journal of clinical practice. 2007;61(11):1905–13. doi: 10.1111/j.1742-1241.2007.01509.x 17655681

[45] Patel KK, Kashfi K. Lipoproteins and cancer: The role of HDL-C, LDL-C, and cholesterol-lowering drugs. Biochemical Pharmacology. 2021:114654. doi: 10.1016/j.bcp.2021.114654 34129857 PMC8665945

[46] McTaggart F, Jones P. Effects of statins on high-density lipoproteins: a potential contribution to cardiovascular benefit. Cardiovascular Drugs and therapy. 2008;22(4):321–38. doi: 10.1007/s10557-008-6113-z 18553127 PMC2493531

[47] Crouse JR Jr, Byington RP, Bond MG, Espefand MA, Craven TE, Sprinkle JW, et al. Pravastatin, lipids, and atherosclerosis in the carotid arteries (PLAC-II). The American journal of cardiology. 1995;75(7):455–9. doi: 10.1016/s0002-9149(99)80580-3 7863988

[48] Bruckert E, Baccara-Dinet M, McCoy F, Chapman J. High prevalence of low HDL-cholesterol in a pan-European survey of 8545 dyslipidaemic patients. Current medical research and opinion. 2005;21(12):1927–34. doi: 10.1185/030079905X74871 16368042

[49] Vaisar T, Pennathur S, Green PS, Gharib SA, Hoofnagle AN, Cheung MC, et al. Shotgun proteomics implicates protease inhibition and complement activation in the antiinflammatory properties of HDL. The Journal of clinical investigation. 2007;117(3):746–56. doi: 10.1172/JCI26206 17332893 PMC1804352

[50] Femlak M, Gluba-Brzózka A, Ciałkowska-Rysz A, Rysz J. The role and function of HDL in patients with diabetes mellitus and the related cardiovascular risk. Lipids in health and disease. 2017;16(1):1–9. 29084567 10.1186/s12944-017-0594-3 PMC5663054

[51] Yadegar A, Mohammadi F, Rabizadeh S, Qahremani R, Esteghamati A, Nakhjavani M. Prevalence of different patterns of dyslipidemia in patients with type 2 diabetes in an Iranian population. Translational Medicine Communications. 2022;7(1):1–8.

[52] Ling J, Koye D, Buizen L, Khunti K, Montvida O, Paul SK. Temporal trends in co‐morbidities and cardiometabolic risk factors at the time of type 2 diabetes diagnosis in the UK. Diabetes, Obesity and Metabolism. 2021;23(5):1150–61. doi: 10.1111/dom.14323 33496366

[53] Li HL, Cheung BM. Trends in Cardiovascular Risk in the United States 1999–2018. Journal of the Endocrine Society. 2021;5(Suppl 1):A422.

[54] Stark Casagrande S, Fradkin JE, Saydah SH, Rust KF, Cowie CC. The prevalence of meeting A1C, blood pressure, and LDL goals among people with diabetes, 1988–2010. Diabetes care. 2013;36(8):2271–9. doi: 10.2337/dc12-2258 23418368 PMC3714503

[55] Li J, Nie Z, Ge Z, Shi L, Gao B, Yang Y. Prevalence of dyslipidemia, treatment rate and its control among patients with type 2 diabetes mellitus in Northwest China: a cross-sectional study. Lipids in Health and Disease. 2022;21(1):1–9. 36002855 10.1186/s12944-022-01691-1 PMC9404639

[56] Rabizadeh S, Mansournia MA, Salehi SS, Khaloo P, Alemi H, Mirbolouk H, et al. Comparison of primary versus secondary prevention of cardiovascular disease in patients with type2 diabetes: focus on achievement of ABC goals. Diabetes & Metabolic Syndrome: Clinical Research & Reviews. 2019;13(3):1733–7. doi: 10.1016/j.dsx.2019.03.043 31235086

